# Pediatric Obesity's Effect on Open and Laparoscopic Appendectomy Outcomes

**DOI:** 10.1055/s-0044-1791968

**Published:** 2024-10-24

**Authors:** Anshul Bhatnagar, Nishtha Nigam, Rohan Anne, Sadashiv Santosh

**Affiliations:** 1 School of Medicine, Baylor College of Medicine, Houston, Texas; 2 Department of Internal Medicine, Monmouth Medical Center, Long Branch, NJ; 3 Department of Neuroscience, Lawrence University, Appleton, Wisconsin; 4 Ascension Columbia St. Mary's Hospital, Mequon, Wisconsin

**Keywords:** laparoscopic appendectomy, open appendectomy, obesity, length of stay, postoperative complications, regression

## Abstract

**Background** Pediatric obesity is a common comorbid condition that may complicate pediatric surgeries, such as appendectomy. Prior research on the consequences of obesity on pediatric appendectomy outcomes have been limited by small-sample sizes and pooled analyses that do not distinguish the effects of surgical approach. Associations between surgical approach, pediatric obesity, and postoperative appendectomy outcomes thus remain unclear.

**Objective** To analyze postoperative appendectomy outcomes by accounting for pediatric obesity, appendectomy approach, and their interaction. This is a retrospective cohort population analysis. Nationwide data of pediatric inpatients from the United States were obtained. All pediatric patients who had an appendectomy were selected from the 2019 Kids' Inpatient Database.

**Materials and Methods** Outcomes variables were the length of stay and postoperative complication rate. The primary exposure variables were pediatric obesity, surgical approach (laparoscopic [LA] vs. open appendectomy [OA]), and an interaction term between the two. Control variables were patient demographics, clinical complexity, and geographic location. Multiple regression was used to determine relationships between the outcome, exposure, and control variables.

**Results** A total of 49,037 pediatric patients had an appendectomy, with the number of OA and LA being 4,517 and 44,420, respectively. LA patients had 5.8% ( *p* < 0.001) shorter length of stay than OA patients. For obese patients, length of stay was 31.8% ( *p* < 0.001) longer than for nonobese patients, but LA (compared with OA) reduced it by 19% ( *p* < 0.007). Obesity had no effect on the number of postoperative complications.

**Conclusion** Obese patients had significantly longer length of stay than nonobese counterparts following OA (31.8%), but this difference was minimized for LA patients (15.81%). There was no association between complication rate, obesity, and surgical approach. Our findings will enable more accurate estimations of pediatric patients' postoperative courses and efficient allocation of limited hospital resources. Further research may wish to study the effects of other common pediatric comorbidities on other procedures.

Acute appendicitis is a common, morbid condition, resulting in 300,000 hospital visits per year in the United States, with the highest incidence in childhood. 1 Although nonsurgical management has been found to be effective in selected patients, surgical appendectomy remains the standard of care. 2 Laparoscopic appendectomy (LA), compared with open surgical approach (OA), is associated with reduced hospital readmission and slightly shorter operating time and hospital length of stay (LOS), but increased rates of intra-abdominal abscess. 3 4

The rate of childhood obesity in the United States has risen significantly in recent decades and been studied extensively. 5 Today, one out of five American children is obese. 6 Prior research has used pooled data or limited analyses to suggest that pediatric obesity worsens outcomes after appendectomy. 7 8 However, prior research is limited for two primary reasons. First, there are two approaches to appendectomy (OA and LA) and analyses that pool both approaches are unable to determine the effects of obesity on each individual surgical approach. Regardless of whether surgeons choose to pursue an open versus laparoscopic approach, physicians will need to provide accurate counseling to obese and nonobese patients regarding postoperative outcomes, and this is not possible given current research. Second, prior research has been limited by small-sample sizes or single-center studies, which may not be representative of the broader U.S. population. 9

In this large database study, we sought to study the effects of pediatric obesity on postoperative appendectomy outcomes, namely LOS and complication. Our novel approach includes both obese and nonobese patients and stratifies by surgical approach to fully determine interactions between obesity, approach, and outcomes. We also use a large pediatric database in the United States to increase our study's generalizability. Our findings will further improve pre- and postoperative patient counseling regarding outcomes. They may also help facility administrators properly budget beds, manage staff, and other limited hospital resources.

## Materials and Methods

### Data Source

This study relied on a retrospective analysis of the Kids' Inpatient Database (KID), the largest all-payer pediatric inpatient database in the United States. Produced every 3 years as part of the national Healthcare Cost and Utilization Project (HCUP), each version of HCUP-KID contains clinical and nonclinical data of over six million weighted pediatric admissions (age < 21 years) from over 4,000 hospitals. The database is designed to produce national estimates. Patients are not followed postdischarge, so readmitted patients may have multiple records. This study used the 2019 version of KID, the most recent one at the time of analysis.

### Patient Selection

Pediatric patients who had an appendectomy were selected from HCUP-KID using the relevant International Classification of Diseases, Tenth Revision (ICD-10) procedure codes (0DTJ0ZZ and 0DBJ0ZZ for OA and all other 0DTJ- and 0DBJ- for LA). Patients were considered obese based on the ICD-10 diagnosis code (E66.XX, except E66.3, Z68.3X, Z68.4X, and Z68.54) from the hospitalization record.

### Outcome Variable

The study's main outcome variables were hospital LOS and number of postoperative complications. The LOS variable was defined as the difference between the inpatient admission and discharge dates; this was log-transformed to account for significant skew. To calculate postoperative complications, we used ICD-10 diagnosis codes to identify patients with any of five common complications: paralytic ileus (prevalence: 10–30%), 10 surgical site infections (prevalence: 6–12%), 11 intra-abdominal abscesses (prevalence: 12%), 12 peritoneal adhesions (prevalence: 54–66%), 13 and other digestive system complications. These postoperative complications were among the most common secondary diagnosis codes associated with appendectomy hospitalizations that were included in our database, hence explaining their selection for study. For each patient, we calculated the postoperative complication rate.

### Exposure Variable

In this study, the surgical approach was either open or laparoscopic. A patient was classified either as obese or nonobese. The third exposure variable was an interaction term between surgical approach and pediatric obesity. This term is used to study how LOS (and postoperative complications) for a particular surgical approach differed between the general and obese population.

### Control Variables

Important control variables included key patient demographics that have been shown to influence the cost of appendectomy, such as age, sex, and median household income for a patient's zip code. 14 The number of procedures for each patient was included to control for the complexity of each diagnosis and the extraneous procedures. Finally, region (split into the four U.S. Census regions) was also included to control for regional differences in outcomes.

### Statistical Analysis

Standard statistical measures (mean, frequency, and percentage) were calculated to describe the patient demographics. To determine how the LOS (and postoperative complication rate) was affected by childhood obesity, surgical approach, and control variables, multiple linear and logistic regression models were used. The sample was weighted using database-provided weights to mirror the national population. The Statistical Package for the Social Sciences (SPSS) version 28.0 was used to conduct all statistical analyses. A two-sided *p* -value of ≤0.05 was considered statistically significant.

## Results

### Descriptive Statistics of Patient Demographics

Table 1 shows the relative frequencies of the key demographics of pediatric patients that had an appendectomy in the United States in 2019. Nationally, 49,037 pediatric patients had an appendectomy. Of these, 44,520 (90.8%) were treated using LA, and 4,517 (9.2%) with OA. Laparoscopic patients were more likely to be obese than open ones (4.2 vs. 2.8%, respectively; *p* < 0.001). The OA patient population was significantly younger and lower income than the laparoscopic population. White patients constituted a majority, followed somewhat closely by Hispanic patients, and then, at a distant third, by Black patients. OA patients had higher costs and longer LOS than LA. Almost 3% ( *n* = 1,297) of all patients experienced a postoperative complication.

**Table 1:** Descriptive statistics about pediatric appendectomy patients

	Open	Laparoscopic	Total	*p* -Value
Median (IQR)				
Age	9 (11)	12 (8)	12 (8)	<0.001
Total hospital charges	13,386 (22,828)	10,385 (6,965)	10,525 (7,475)	<0.001
Length of stay	4 (8)	2 (3)	2 (3)	<0.001
Count/frequency, % ( *n* )				
Comorbidity				
Obese	2.8 (129)	4.2 (1,890)	4.1 (2,018)	<0.001
Nonobese	97.2 (4,388)	95.8 (42,631)	95.9 (47,019)	
Sex				
Male	39.9 (1,802)	59.6 (26,540	59.7 (29,253)	0.535
Female	60.1 (2,713)	40.4 (17,980	40.3 (19,782)	
Median household income of patient's zip code				
1st quartile ($1–$47,999)	34.0 (1,520	29.1 (12,799)	29.5 (14,319)	<0.001
2nd quartile ($48,000–$60,999)	26.5 (1,183)	24.4 (10,715)	24.5 (11,898)	
3rd quartile ($61,000–$81,999)	22.8 (1,019)	25.0 (11,012)	24.8 (12,0332)	
4th quartile ($82,000 + )	16.7 (746)	21.5 (9,476)	21.1 (10,222)	
Region				
Northeast	18.5 (834)	15.5 (6,914)	15.8 (7,748)	<0.001
Midwest	20.4 (923)	14.8 (6,596)	15.3 (7,520)	
South	37.0 (1,672)	35.0 (15,579)	35.2 (17,250)	
West	24.1 (1,087)	34.7 (15,432)	33.7 (16,519)	

Abbreviation: IQR, interquartile range.

Notes: The numbers are percentages. The numbers in brackets are the actual frequency count.

### Obesity's Association with Length of Stay

Table 2 shows associations between obesity, surgical approach, and LOS. Since the dependent variable is measured in log, the regression coefficient β is converted into percentage probability by using the Eq. 100*(exp(β) − 1). Obese patients had approximately 31.8% (β = 0.276, *p* < 0.001) longer hospitalizations than nonobese patients. LA patients had significantly shorter hospitalizations approximately 5.8% (β = − 0.056, *p* < 0.001) less than that of OA patients. This effect was magnified for obese patients. Obese patients who had LA experienced 19% (β = − 0.174, *p* < 0.007) shorter hospitalizations compared with those who received OA. Older patients, females, and those living in higher-income areas had significantly shorter hospitalizations. Appendectomy patients living in the West had the shortest hospitalizations, followed by those in the Northeast, Midwest, and South.

**Table 2:** Multivariate regression results for length of stay and postoperative complication rate

	Length of stay a				Postoperative complication rate		
	Coefficient	95% CI	*p* -Value	% change	Odds ratio	95% CI	*p* -Value
Age	−0.035	(−0.04, −0.03)	<0.001	−3.44	0.999	(0.99, 1.01)	0.834
Sex							
Male	Ref	–	–		Ref	–	–
Female	−0.014	(−0.03, 0)	0.033	−1.39	0.646	(0.57, 0.73)	<0.001
Median household income of patient's zip code	−0.022	(−0.03, −0.02)	<0.001	−2.18	1.024	(0.97, 1.08)	0.362
Number of procedures	0.194	(0.19, 0.2)	<0.001	21.41	1.168	(1.15, 1.19)	<0.001
Region							
Northeast	Ref	–	–		Ref	–	–
Midwest	0.099	(0.08, 0.12)	<0.001	10.41	1.299	(1.06, 1.59)	0.012
South	0.110	(0.09, 0.13)	<0.001	11.63	1.319	(1.1, 1.58)	0.002
West	−0.074	(−0.09, −0.05)	<0.001	−7.13	1.011	(0.84, 1.22)	0.910
Approach							
Open	Ref	–	–		Ref	–	–
Laparoscopic	−0.056	(−0.08, −0.03)	<0.001	−5.45	1.021	(0.83, 1.25)	0.839
Comorbidity							
Nonobese	Ref	–	–		Ref	–	–
Obese	0.276	(0.15, 0.4)	<0.001	31.78	1.680	(0.75, 3.76)	0.207
Interaction term							
LA a obesity	−0.174	(−0.3, −0.05)	0.007	−15.97	0.744	(0.32, 1.74)	0.496

Abbreviations: CI, confidence interval; LA, laparoscopic appendectomy; Ref, reference.

a Length of stay = ln (number of days).

### Obesity's Association with Postoperative Complications

Table 2 shows associations between obesity, surgical approach, and the number of postoperative complications. There was no significant interaction between obesity and surgical approach, and neither variable was associated with postoperative complication rate. Female sex was associated with significantly lower odds of postoperative complications. Patients in the Midwest and South had significantly higher odds of experiencing postoperative complications. Neither age nor income was associated with postoperative complication rate.

## Discussion

Our findings suggest that pediatric obesity significantly increases LOS for both open and laparoscopic patients, but this effect varies by surgical approach. For patients undergoing OA, obesity increases the LOS by 31.78%. However, for patients undergoing LA, obesity increases the LOS by only 15.81%. We did not find any effect of pediatric obesity or surgical approach on the postoperative complication rate. These are new findings. Although the decision to pursue LA versus OA is made based on patient characteristics and disease complexity, providers and parents of obese children will wish to be aware of how this comorbidity affects outcomes following each individual approach. While previous studies using pooled analyses have shown that pediatric obesity has negative impact on the LOS following appendectomy in general, we argue against using such data, as this provides less detailed and accurate predictions of patients' postoperative courses. We show that the LOS varies with the appendectomy approach, pediatric obesity, and their interaction. Therefore, while predicting the LOS for appendectomy patients, the patient's obesity and appendectomy approach should both be factored in the model.

Our study has both practical and academic implications. Practically, these findings will be helpful for hospital administrators who are interested in optimal utilization of limited hospital resources, such as hospital beds and nursing and other ancillary staff. An important input in these calculations is how long patients recovering from surgeries need to stay in the hospital 15 and the likelihood of postoperative complications, which may require additional resources and care. For example, we find that younger pediatric patients with appendicitis have longer hospitalizations, a finding supported by prior research that suggests younger patients are more likely to experience perforation, postsurgical complications, and misdiagnosis rates. 16 17 This may be explained by younger patients' reduced ability to verbally communicate symptoms with health care providers. We also found that appendectomy patients in the Midwest and South were more likely to have prolonged hospitalizations and postsurgical complications compared with patients in the Northeast or West. This is supported by prior research among pediatric appendectomy patients in the United States, which suggests increased rurality in the Midwest and South may lead to increased travel times to medical centers and an increased likelihood of perforation on presentation. 18 More accurate and specialized predictions of postoperative courses following each individual surgical approach may enable more efficient use of limited health care resources.

Our study can also help appendectomy providers make better predictions about postoperative hospitalization courses for each individual patient and enable more accurate counseling for patients' families based on specific patient comorbidities. For example, parents of an appendectomy patient will often need to predict time away from work and home and organize childcare or eldercare for other dependents. Therefore, an accurate understanding of their child's postoperative course based on all pertinent factors, such as choice of surgical approach, comorbid obesity, and other characteristics, is critical for parents. 19 The findings discussed in this study will enable doctors to make more accurate estimates of the LOS and complication rate that they can provide to both their nonobese and obese patients. Further research may wish to use a similar stratified approach to understand the effects of other common pediatric comorbidities that may complicate surgery.

Further research may wish to use a similar stratified approach to understand the effects of other common pediatric comorbidities that may complicate surgery. The methods employed in this study can be used to examine how other common pediatric comorbidities, such as diabetes, chronic pulmonary diseases, and fluid and electrolyte disorders, influence the outcomes of common pediatric surgeries. The valuable information gained from such studies can thus be used alongside other metrics to provide better estimates of LOS, as well as other surgical outcomes, such as hospital costs and the postoperative complication rate.

The HCUP-KID data, like all large nationwide administrative databases, is not without its limitations. This database relies heavily on proper identification of the relevant ICD-10 diagnosis and procedure codes, which are sometimes missing or incorrect. 20 This can lead to an underrepresentation of pediatric obesity within the database, leading to possible selection bias. Additionally, some clinical factors like postoperative pain, readmission rates, and long-term outcomes are not available in HCUP-KID, leading to possible omitted-variable bias, and consequently our study could not consider these important metrics. Despite some drawbacks, this study provides more accurate and individualized estimates of postoperative outcomes for both obese and nonobese patients following either an OA or LA.

## Conclusion

We found that the LOS following appendectomy depends on the surgical approach, pediatric obesity, and their interaction. Specifically, for patients undergoing OA, obesity increases the LOS by nearly one-third. However, for patients undergoing laparoscopic obesity, the increase is only one-sixth. Our findings may foster improved provider–patient pre- and postoperative counseling and enable optimal allocation of scarce hospital resources, such as beds and nursing staff. Obese and nonobese appendectomy patients and their families will also benefit from more accurate predictions about postoperative hospitalization courses. Further research may wish to use a similar stratified approach to study the effects of other common pediatric comorbidities on the outcomes of other procedures.
